# A Notable Prize Essay

**Published:** 1907-05

**Authors:** 


A NOTABLE PRIZE ESSAY.—We have before mentioned Dr. S. A. Knopf's prize essay on tuberculous disease, but the recent appearance of a fourth edition (New York, 1907) makes it only due to the distinguished author that we should call attention anew to the extraordinary degree to which he has impressed the medical profession and the world. It may well be a matter of pride with us that this New York physician has met with such great success in enlisting general sympathy in the campaign against consumption. The prize was awarded by the International Congress to Combat Tuberculosis as a Disease of the Masses, which held its session in Berlin in May, 1899.

Dr. Knopf's masterly essay was written in German, and has been translated (by himself) into English (with an edition, by Dr. J. M. Barbour, adapted to British use), Arabic, Portuguese, Bulgarian, Dutch, Finnish, French, Hebrew, Hungarian, Icelandic, Italian (two translations), Japanese, Spanish (with an additional translation for Mexico), Polish, Russian (two translations), Servian, Swedish, and Turkish. If there are any other medical essays that have met with such widespread appreciation in recent years, we have yet to hear of them. Dr. Knopf had something tangible to say, and he said it in plain and simple words. He dealt with the problems of tuberculous disease from the common sense point of view. Those, as it seems to us, are the reasons why his essay has been so universally acclaimed.

Dr. Knopf—and this is in the highest degree to his credit—has not been content to enjoy the honors brought to him by his prize essay; he has continually bestirred himself in the interest of the consumptives by addresses, journal articles, and personal work.—*New York Medical Journal.*

